# FGF coordinates air sac development by activation of the EGF ligand Vein through the transcription factor PntP2

**DOI:** 10.1038/srep17806

**Published:** 2015-12-03

**Authors:** Josefa Cruz, Neus Bota-Rabassedas, Xavier Franch-Marro

**Affiliations:** 1 Institute of Evolutionary Biology (IBE, CSIC-Universitat Pompeu Fabra), P. de la Barceloneta 37, 08003 Barcelona, Catalonia, Spain

## Abstract

How several signaling pathways are coordinated to generate complex organs through regulation of tissue growth and patterning is a fundamental question in developmental biology. The larval trachea of *Drosophila* is composed of differentiated functional cells and groups of imaginal tracheoblasts that build the adult trachea during metamorphosis. Air sac primordium cells (ASP) are tracheal imaginal cells that form the dorsal air sacs that supply oxygen to the flight muscles of the *Drosophila* adult. The ASP emerges from the tracheal branch that connects to the wing disc by the activation of both Bnl-FGF/Btl and EGFR signaling pathways. Together, these pathways promote cell migration and proliferation. In this study we demonstrate that Vein (*vn*) is the EGF ligand responsible for the activation of the EGFR pathway in the ASP. We also find that the Bnl-FGF/Btl pathway regulates the expression of *vn* through the transcription factor PointedP2 (PntP2). Furthermore, we show that the FGF target gene *escargot (esg)* attenuates EGFR signaling at the tip cells of the developing ASP, reducing their mitotic rate to allow proper migration. Altogether, our results reveal a link between Bnl-FGF/Btl and EGFR signaling and provide novel insight into how the crosstalk of these pathways regulates migration and growth.

Signaling pathways regulate many processes underlying organogenesis, such as patterning, cell division and growth. However, in order to form a fully functional organ, signaling pathways need to be synchronized during development in a very precise way. One process that requires the coordinated orchestration of multiple signaling pathways is Tubulogenesis. This process underlies the formation of organs such as the mammalian lung, kidney, mammary gland or the insect tracheal system. However, the spatial and temporal regulation of these signaling pathways is poorly understood. Here we demonstrate that the fibroblast growth factor (Bnl/FGF) pathway plays a key role in the regulation of epidermal growth factor (EGFR) signaling during the development of the tracheal system of the adult fly.

During *Drosophila* metamorphosis the tracheal system undergoes an extensive remodeling to give rise to the new respiratory structures in the adult organism, such as the air sacs of the dorsal thorax. These structures are dilations of the main tracheal trunks that work as air reservoirs in the adult^1^,^2^. Air sac development is particularly interesting, as it resembles vertebrate lung and vascular organ formation, where proliferation and cell death are concomitant with branching morphogenesis. The air sac primordium (ASP) develops during early third larval instar (L3) from a tracheal transverse connective branch (TC) close to the wing disc, where a group of imaginal tracheoblasts responds to the expression of the FGF ligand Branchless (Bnl). Bnl is secreted in the underlying wing disc cells, and activates the FGF receptor Breathless (Btl) in the ASP cells. As the expression of Bnl in the wing disc changes, the ASP migrates and grows in direction of the source^1^.

While Bnl/FGF signaling controls ASP migration, EGFR regulates cell proliferation and cell survival^1^,^3^. Bnl/FGF signaling activity is restricted to the migrating tip cells, the cells of the distal/middle part of the ASP, where it activates the transcription of several genes, such as the transcription factor Esg and matrix metalloprotease Mmp2. While Esg is essential for tip cell identity, Mmp2 has two important functions in ASP development. Firstly, Mmp2 regulates the levels of Collagen IV and Perlecan in the extracellular matrix surrounding disc-associated trachea and ASP, in order to align juxtaposed tracheal and disc cells^4^. Secondly, it represses the activation of Bnl/FGF signaling in the stalk cells, the tracheoblasts of the proximal/middle part of ASP^5^. In contrast to Bnl/FGF signaling, the EGFR pathway is activated all along the ASP promoting cell proliferation. However, EGFR pathway seems to be activated to higher levels in the stalk/middle cells inducing more cell divisions in this specific region of the ASP^3^. Despite these studies, a number of important questions remain elusive: which ligand is responsible for EGFR activation? how it is related to restricted Bnl signaling? and how ASP migration and growth are globally coordinated?

Here we show that the Bnl/FGF pathway coordinates ASP growth by the activation of the EGF ligand Vn expression through the transcription factor Pointed (Pnt). Our genetic analysis reveal that Vn is the main EGF ligand in the stalk cells of the ASP, as silencing of *vn* abrogates the formation of this organ. In addition we find that the transcription factor *esg*, a FGF target gene, which is highly expressed at the tip cells, attenuates EGF signaling and thus cell proliferation in this region of the ASP. Importantly, this study reveals a link between Bnl/FGF and EGFR pathways during ASP development and provides new insight into how these signaling pathways coordinate to control cell migration and tissue growth.

## Results

### Vn is the EGFR ligand in the ASP

**Figure 1:**
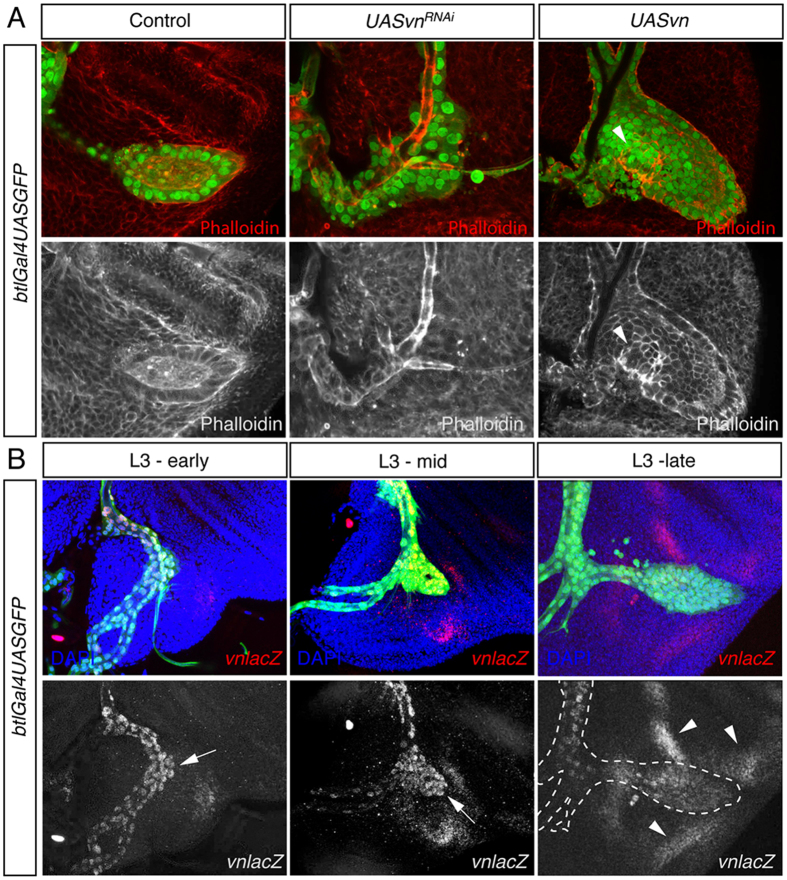
Vn is the main ligand of EGFR pathway in the ASP. (**A**) *vn* expression was either silenced or over-expressed in the tracheal cells using *btlGAL4UASnsGFP* (green). Expression of *vn*^*RNAi*^ abolished the formation of ASP. In contrast over-expression of Vn increased dramatically the number of tracheoblasts of the ASP forming bulgy outgrowths. Phalloidin marked the cell morphology of ASP tracheoblasts. Note the bulge produced by *vn* over-expression (arrowhead). (**B**) Expression of *vn* using a *vnlacZ* enhancer trap in the ASP in early, mid and late L3 instar larva. In early L3, *vnlacZ* (red) was detected in the tracheal branch attached to the wing disc with higher levels at the tip of the incipient ASP (arrow). In mid L3 *vn* expression was even stronger among the tip/middle cells (arrow). Finally in late L3 instar expression of *vn* was more enriched in the stalk cells. Note that *vn* was also detected in the disc areas surrounding the ASP (arrowhead).

*Drosophila* has four EGFR ligands: Spitz (Spi), Keren (Krn), Gurken (Grk) and Vn. Vn is produced as a mature secreted protein, while the other three ligands require maturation by the protease Rhomboid (Rho)^6^. *grk* is a specific ligand of EGFR in oogenesis^7^ and *Krn* has a minor role as homozygous *Krn* mutant flies are viable^8^. Therefore, to identify which of the *Drosophila* EGFR ligands is responsible for EGFR pathway activation in the ASP we disrupted *spi* and *vn* gene function in the tracheal system of third instar larvae. This was achieved by the expression of specific RNAi constructs for either gene, using the trachea-specific *btlGal4* driver, and controlled temporally through the co-expression of a temperature-sensitive Gal80^ts^ suppressor. The efficacy of the RNAi-lines was validated in the wing disc where a typical EGFR loss of function phenotype can be easily observed (Supplementary Fig. 1A–D). Knockdown of *spi* had no effect on either adult wing^9^ or ASP formation (Supplementary Fig. 1A,C). Consistent with this result, overexpression of Argos (Aos), which inhibits EGFR signaling^10^ through the specific capturing of the Spi ligand^11^, also showed no phenotype in air sac development (Supplementary Fig. 1D). In addition, using an antibody against Spi and a *rholacZ* reporter strain as a read out of the activity of Spi, we did not detect any Spi protein, and only a very weak expression of *rholacZ* in the ASP (Supplementary Fig. 1E,F). In contrast, depletion of *vn* by RNAi produced a strong impairment in air sac formation (Fig. 1A and Supplementary Fig. 1B). Consistently, over-expression of *vn* specifically triggered a higher cell division rate, generating bulgy outgrowths, without perturbing air sac guidance (Fig. 1A, arrowhead). These results strongly suggest that *vn* is the ligand responsible of the EGFR pathway activation during air sac development.

Next we checked the expression pattern of *vn* in the developing ASP. Immunostaining against β-galactosidase of *vn-lacZ* reporter and GFP expression under the control of *vnGal4* revealed *vn* expression in the ASP cells (Fig. 1B and Supplementary Fig. 2). The expression of *vn* was already detected at early L3 in all tracheal cells of TC branch, when the ASP starts budding. As the ASP formed and migrated across the wing imaginal disc *vn* expression increased in those cells (Fig. 1B and Supplementary Fig. 2), suggesting that *vn* expression is regulated in the ASP cells.

In addition to *vn* expression in the tracheal cells, *vn* was also detected in the wing disc cells adjacent to the ASP (arrowheads in Fig. 1B and Supplementary Fig. 2). Since Vn is a secreted protein, it might be that *vn* expression in the wing disc exerts an effect on the ASP in a non-autonomous manner. To assess this possibility we knocked down *vn*, using *apterousGal4* driver, which is restricted in the dorsal compartment of the wing disc where the ASP forms. Interestingly, depletion of *vn* in the dorsal compartment of the wing disc had no effect on ASP development, proving that the specific expression of *vn* in the ASP is responsible for EGFR activation during the development of this structure (Supplementary Fig. 1G).

### Vn promotes proliferation and cell survival during air sac development

**Figure 2:**
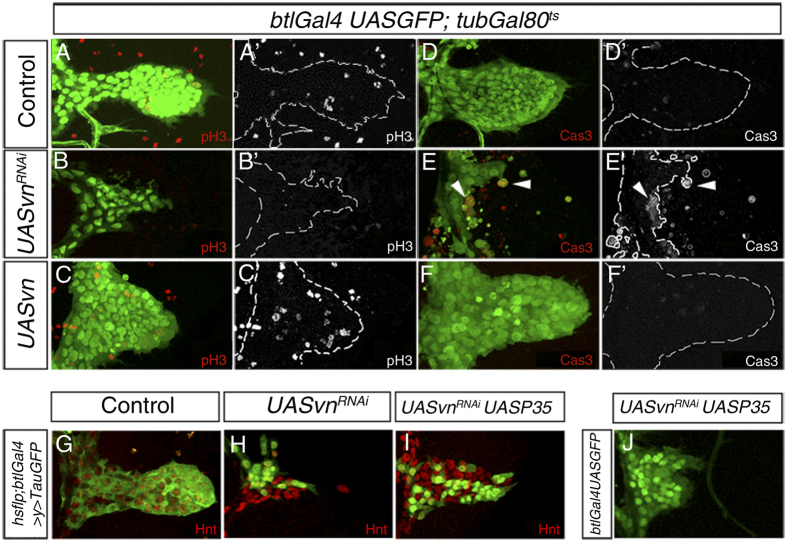
Vn promotes cell proliferation and cell survival during ASP development. (**A–F’**) Third instar larvae of the genotype *btlGAL4UASnGFP*; *tubGAL80*^*ts*^ (in green) were used to visualize tracheal cells. The air sacs are outlined with the dotted line. Anti-Phosphohistone H3 antibody (in red) marked proliferating cells of the outgrowing air sac (**A,A’**). No PH3 positive cells in ASP expressing *vn*^*RNAi*^ were detected (**B,B’**). In contrast, ASP expressing Vn ectopically presented more mitotic cells (**C,C’**). (**D–F’**) Anti Caspase3 antibody was used to detect cell death. Cas3 positive cells were detected in neither control nor ASP over-expressing Vn (**D,D’,F,F’**). In contrast, multiple dying cells were visible in ASP expressing *vn*^*RNAi*^ (**E,E’**) (arrowheads). (**G–I**) Flip out clones expressing only *tauGFP* (**G**), *vn*^*RNAi*^ (**H**) or *vn*^*RNAi*^ and *UASp35* (**I**) to prevent apoptosis. Air sacs were marked with Hnt antibody (red). Note the expansion of the clones found in the ASP (compare **H** and **I**). (**J**) Air sac expressing *UASp35* in a *vn*^*RNAi*^ mutant under the control of *btlGAL4UASGFP*. Note that apoptosis inhibition rescues partially ASP size.

The fact that the lack of *vn* strongly disrupted the formation of the ASP prompted us to investigate whether this phenotype is due to either an increase in cell death, or a reduction in cell proliferation. Under wild-type conditions proliferating ASP cells were detected using Phosphohistone-3 (PH3) a marker often used to identify dividing cells^12^ (Fig. A,A’) whereas apoptosis was monitored by cleaved Caspase-3 staining, which was not detected (Fig. 2D,D’). In contrast, expressing *vn*^*RNAi*^ throughout the tracheal system using *btlGal4* caused a drastic reduction specifically in the ASP, in proliferative cells (Fig. 2B,B’) and an increase in cell death (Fig. 2E,E’). This effect is specific of the ASP cells, as the other mitotic populations of the tracheal system are unaffected (Supplementary Fig. 3). Consistently, over-expression of Vn in ASP tracheal cells using the same driver increased cell division (Fig. 2C,C’) without affecting apoptosis (Fig. 2F,F’). Interestingly, co-expression of *vn*^*RNAi*^ and P35, a viral inhibitor of apoptosis^13^, in *flip out* clones increased the size of *vn* depleted clones (Fig. 2G–I) and partially restored the number of tracheoblasts in the ASP when co-expressed in all tracheal cells (Fig. 2J). These results suggest that loss of tissue in the absence of *vn* is caused by both a reduction in cell proliferation and an increase in cell death, supporting the previously reported role of EGFR pathway in the growing ASP^3^. Thus, we conclude that Vn activates the EGFR pathway to sustain the rate of proliferation that normally takes place during ASP development and to maintain cell viability.

### Bnl/FGF signaling activates Vein expression in the ASP

**Figure 3:**
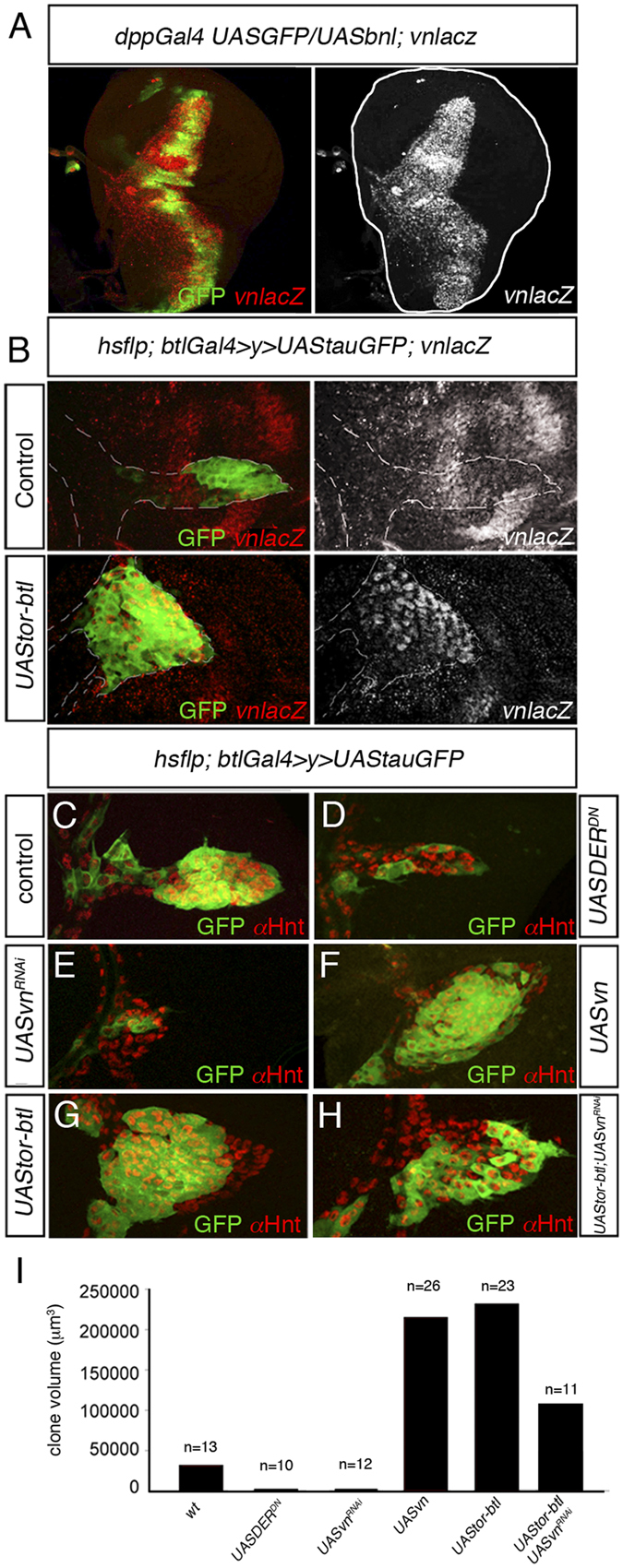
Bnl-FGF/Btl signaling induces *vn* expression in ASP. (**A**) Ectopic *bnl* expression (*dppGAL4UAS-GFP; UAS-bnl*) (green) in the wing disc induced new tracheoblasts expressing high levels of *vn* (red). (**B**) *vnlacZ* expression (red) in random flip out clones in the tracheal system expressing either *tauGFP* or constitutively active Btl (Tor-Btl). Note that the over-activation of FGF/Btl signaling in ASP increased the number of tracheoblasts and the levels of *vnlacz.* (C-H) Flip out clones generated in the tracheal system expressing *UAStauGFP* (**C**), *UASDER*^*DN*^ (**D**), *UASvn*^*RNAi*^ (**E**), *UASvn* (**F**), *UAStor-btl* (**G**) and *UAStor-btl* and *UASvn*^*RNAi*^ (H). Note the reduction of clone size in (**H**) compared to (**G**). (**I**) Quantification of clone volume. Note that overexpression of *vn*^*RNAi*^ reverted the overgrowth produced by the over-activation of FGF/Btl signaling. The number of clones analyzed is indicated in the histogram.

Ectopic activation of Bnl/FGF signaling causes a significant increase in the number of ASP tracheoblast cells^1^. However, a previous report suggested that the essential role of FGF signaling is to control directed organ extension via cell migration, whereas it is EGFR signaling that promotes cell proliferation^3^. Such observations prompted us to hypothesize that Bnl/FGF signaling might regulate EGFR signaling by controlling the expression of *vn* in the ASP. To investigate this possibility, we ectopically expressed Bnl/FGF in the wing disc using *dppgal4* driver. Under this condition, multiple tracheoblasts were generated and migrated over the wing disc epithelium along the region of *bnl* expression (Fig. 3A and Supplementary Fig. 4A,B). Interestingly, *vn* expression was detected in all ectopic tracheoblasts (Fig. 3A and Supplementary Fig. 4A,B) suggesting that Bnl/FGF signaling controls *vn* expression in the ASP. Accordingly, similar results were obtained when over-expression clones of a constitutively activated form of Btl receptor (Torso-Btl/Fgfr) were generated in the ASP. Tracheoblasts with over-activated Bnl/FGF signaling expressed higher *vn* levels and therefore proliferated at a higher rate (Fig. 3B). Similar over-proliferation phenotype was obtained when Bnl/FGF was expressed in all ASP cells (Supplementary Fig. 4C,D).

To confirm that the overgrowth produced by ectopic expression of the Btl receptor activated form was due to a *vn* up-regulation, we compared loss and gain of function mutant clones of EGFR signaling and Bnl-FGF/Btl signaling and measured the resulting clones volume. Compared with the control, both an EGFR dominant negative form and *vn*^*RNAi*^ over-expression reduced clone size significantly (Fig. 3C–E,I and Supplementary Fig. 5A–C). In contrast, larger clones were observed after over-expression of either Torso-Btl/Fgfr or Vn protein (Fig. 3F,G,I and Supplementary Fig. 5E,F). Finally, as expected, co-expression of Torso-Btl/Fgfr and *vn*^*RNAi*^ reduced clone size to levels similar to control (Fig. 3H,I). Thus, we conclude that Bnl-FGF/Btl signaling activates tracheoblast proliferation and acts to maintain cell viability by activating EGFR signaling through the regulation of *vn* expression.

### Bnl-FGF/Btl signaling regulates *vn* expression via Pnt

**Figure 4:**
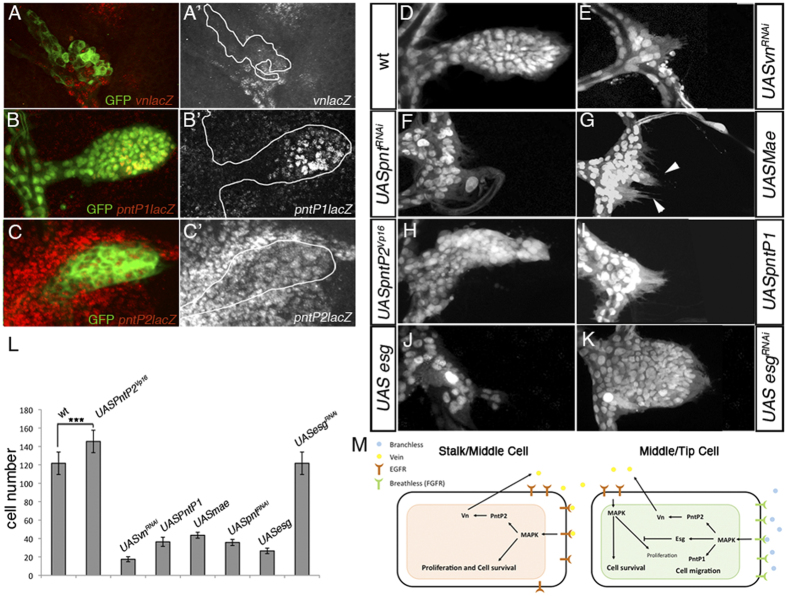
Role of PntP2 in the induction of *vn* expression. (**A–A’**) Random clone expressing *pnt*^*RNAi*^ in the ASP. Expression of *vnlacz* (red) was abolished in *pnt*^*RNAi*^ mutant clones. Clones are visualized by the expression of GFP. Clone shape is outlined with the white line. (**B–C’**) Expression pattern of the two *pnt* isoforms, PntP1 and PntP2 in the ASP cells visualized by *btlGAL4UASGFP*. Enhancer trap *pntP1lacz* was expressed in the distal middle part of ASP (**B,B’**) whereas *pnP2lacz* was detected in all ASP cells (**C–C’**). (**D–K**) ASP expressing different constructs under the control of *btlGAL4UASnGFP; tubGAL80*^*ts*^. Wild type ASP (**D**). Knockdown of *vn* (**E**) and *pnt* (**F**) in the ASP abolished ASP formation. Ectopic expression of *UASmae*, a specific inhibitor of PntP2 isoform, abrogated ASP development but did not affect the capacity of the formation of filopodia (arrowheads) in tracheoblasts (**G**). Expression of an activated form of PntP2 (*UASpntP2*^*VP16*^) enlarged ASP (**H**) whereas ectopic expression of *UASPntP1* abrogated cell proliferation (**I**). Forced expression of *esg* in the ASP reduced dramatically the number of tracheoblasts (**J**). In contrast expression of *esg*^*RNAi*^ resulted in a more rounded ASP by increasing the number of cells at the tip (K). (**L**) Mean cell number of ASP in *btlGAL4UASGFP* (control) or *btlGAL4UASGFP UASpntP2*^*Vp16*^*, UASvn*^*RNAi*^*, UASpntP1, UASmae, UASpnt*^*RNAi*^*, UASesg and UASesg*^*RNAi*^. Statistical significance was assessed with the Student’s t test. ***P < 0.001; n > 10. Error bars indicate the SD. (**M**) Outgrowth of the air sac precursors requires the concomitant activation of two RTKs signaling pathways. Bnl-FGF/Btl is activated at the distal/middle part of the growing air sac, whereas EGFR signaling is activated in all areas of the ASP. Bnl-FGF/Btl signaling triggers cell migration at the tip of the growing structure via PntP1, and initiates the up-regulation of *vn* through PntP2. Vn in turn activates EGFR signaling promoting cell division and cell survival in the ASP. Bnl-FGF/Btl target gene Esg attenuates the action of EGFR signaling at the tip restricting the number of cells that actively migrate. In the stalk/middle cells, autocrine Vn/EGFR signaling establishes a positive feedback loop that maintains *vn* expression in all ASP cells.

To further understand how Bnl-FGF/Btl signaling regulates *vn* expression we investigated the role of Pnt, an ETS domain transcription factor that mediates Ras/Map kinase signaling^3^,^14^,^15^. The Drosophila *pnt* gene encodes two ETS proteins, PntP1 and PntP2, which are controlled by two distinct promoters^16^. PntP1 and PntP2 share a conserved C-terminal DNA-binding ETS domain but PntP2 contains an additional Sterile Alpha Motif (SAM)/PNT domain, which is phosphorylated by MAPK^15^. Consequently upon activation of the MAPK signaling cascade, PntP1 functions as a constitutively active transcription factor, whereas PntP2 protein induces transcription only when phosphorylated^15^. Interestingly, phosphorylated PntP2 binds directly to the *vn* regulatory region controlling its expression pattern in early wing development^17^. To test whether PntP2 and PntP1 also regulate *vn* expression in the ASP, we generated *pnt* knockdown clones in the ASP using two independent *pnt*^*RNAi*^ lines that deplete both isoforms (Supplementary Fig. 6). In *pnt* depleted cells *vn* expression was abolished (Fig. 4A,A’) and consistently the formation of the ASP was prevented in a similar way to *vn*^*RNAi*^ condition (Fig. 4E,F). In addition, when *pnt*^*RNAi*^clones are generated in the ASP, we mainly recover only small sized clones (Supplementary Fig. 4D). Next we checked the *pnt* expression pattern in the ASP. Using PntP1 and PntP2 specific *lacZ* reporter lines^14^,^18^, we found that these isoforms show different expression patterns in the ASP. Whereas PntP1 presented a more enriched expression at the distal/middle region of the ASP (Fig. 4B,B’;^19^) PntP2 is expressed in all ASP cells (Fig. 4C,C’ and Supplementary Fig. 7), suggesting distinct roles for Pnt isoforms in *vn* regulation. To confirm this possibility we ectopically expressed a specific inhibitor of PntP2, an ETS-domain lacking edl/mae^20^ in all the ASP tracheal cells. Inhibition of PntP2 strongly impaired ASP development, although the cells still projected multiple filopodia (Fig. 4G, arrowheads). Consistently, over-expression of a PntP2 activated form PntP2^VP16^ 21, which should circumvent the need for upstream pathway components, significantly increased the size of ASP (Fig. 4H,L). On the other hand, over-expression of PntP1 in the ASP blocks cell proliferation, probably by conferring migratory features on all tracheal cells (Fig. 4I,L). Altogether, these data suggest that Bnl-FGF/Btl signaling controls cell migration of the ASP by the up-regulation of PntP1, and *vn* expression through PntP2.

### FGF attenuates EGFR activity at the tip cells of the ASP

Although cell divisions in the ASP occur in both the proximal and distal regions at similar rates, the central region of the sac presents twice the number of cells than the tip region^3^. A distinct activity of EGFR signaling in the central region versus the tip region might explain the difference in regional cell number. Since Bnl-FGF/Btl signaling is only activated at high levels in cells at the tip^3^ it is reasonable to assume that this pathway might reduce the activity of EGFR in this region. To assess this possibility, using *btlGal4/tubGal80^ts^* we ectopically expressed Esg in the tracheal system. Esg is a transcription factor regulated by Bnl-FGF/Btl signaling and mediates tip-cell-specific functions in the developing tracheal system^1^,^5^,^19^,^22^,^23^. Interestingly, this manipulation caused a dramatic reduction in cell number (Fig. 4J,L). Conversely, *esg* silencing using *esg*^*RNAi*^ under the control of *btlGal4/tubGal80^ts^*, produced a significant increase in PH3 positive cells at the tip/middle part of the ASP, thus expanding the cell number of this area and modifying the ASP morphology (Fig. 4K and Supplementary Fig. 8B–D). Altogether our results indicate that *esg* might alleviate EGFR signaling activity at the tip of the ASP. Nevertheless, our data do not discard completely the possibility that Esg could attenuate cell proliferation in an independent EGFR/Vn mechanism. Yet, we support the idea that Bnl-FGF/Btl signaling exerts a mitogen effect in the ASP by modulating the activity of EGFR signaling, activating the pathway through the up-regulation of its ligand Vn and attenuating it through the expression of the transcriptor factor Esg.

## Discussion

Two receptor tyrosine-kinases (RTKs), Btl/FGFr and EGFR have been implicated in the control of ASP growth and migration along the underlying wing imaginal disc. While Bnl-FGF/Btl signaling is required for directional cell migration, EGFR signaling is needed for cell division and survival^3^. In this study we have identified the mechanism through which these two signaling pathways are coordinated to develop the functional ASP.

We identified Vn as the ligand responsible for the activation of EGFR signaling during ASP development, while the other EGFR ligands seem to play either no role, or only a residual one. We have also shown that Bnl-FGF/Btl signaling induces the expression of *vn,* as over-activation of the pathway produces overgrowth by up-regulating *vn* expression, and such overgrowth is reverted when *vn* is silenced (Fig. 3G,H). Therefore we propose a model where Bnl-FGF/Btl signaling activates EGFR signaling by regulating *vn* expression (Fig. 4M). However, high Bnl-FGF/Btl signaling occurs only at the tip of ASP^1^,^3^,^5^, whereas *vn* expression is detected in all ASP tracheal cells. How then, is *vn* expression maintained outside the Bnl-FGF/Btl domain? In the embryo and the wing disc *vn* is also a target of EGFR signaling through Pnt, forming a feedback loop that regulates EGFR signaling^24^,^25^,^26^. In the ASP a similar process might also apply for regulation of *vn* expression. Thus at early L3 Bnl-FGF/Btl signaling is activated in a subset of tracheal cells that up-regulate *vn* expression. As the growth of the ASP proceeds and the stalk cells move away from the Bnl-FGF/Btl activation domain, EGFR signaling might provide the input to maintain *vn* transcription in those cells in a positive feedback loop. Interestingly, a similar process has recently been reported in the wing disc where once initiated, *vn* expression is amplified and maintained by autocrine signaling mediated by PntP2^17^.This is consistent with our finding that *vn* induction in the ASP is mainly mediated by the transcription factor PntP2, which is likely through direct binding as multiple putative ETS binding sites have previously been identified in the *vn* promotor^17^. In addition, our analyses show that Pnt has an active role in proliferation since most *pnt*^*RNAi*^ mutant clones generated in the ASP are very small (Supplementary Fig. 5D). Interestingly, PntP2 is expressed in all ASP cells and specifically blocking this isoform in the ASP impairs cell proliferation but not cell migration, as tracheal cells still emit filopodia (Fig. 4G). Moreover, over-expression of a specific PntP2 isoform induces a bigger ASP with more cells (Fig. 4H,L). Consistently, the other isoform PntP1 is only expressed in the distal part of the ASP, and its over-expression blocks cell proliferation, most likely by conferring migratory features on all cells (Fig. 4I). These differing functions of *pnt* isoforms could explain the different output of the activation of those two different RTKs in ASP. Thus, whereas both Bnl-FGF/Btl and EGFR signaling are able to activate PntP2 through its phosphorylation, only Bnl-FGF/Btl signaling is able to up-regulate the constitutive activator of transcription PntP1, which in turn activates genes involved in migration (Fig. 4M). However, it has been shown that Pnt seems to be required only for cell migration but not for cell proliferation^3^. A possible explanation for such a discrepancy would be that the *pnt* alleles used in this study only partially eliminate PntP2, as previously suggested^27^.

We also showed that up-regulation of *vn* activates EGFR signaling in all ASP cells, promoting cell division (Fig. 2C,C’). However, the number of cell divisions in the ASP is not homogenous, with twice as many in the central part in contrast to the most distal part. This observation suggested a possible regional restriction on EGFR activity^3^. In this regard we provide evidence that the transcription factor *esg* restricts the EGFR response in the tip cells. *esg* is induced exclusively in tracheal cells that receive FGF/Btl signal, giving those cells a tip-cell fate^1^,^5^,^19^,^22^,^23^. When *esg* is silenced the ASP has a more rounded shape, due to an increase in cell division in the tip cells, without affecting cell viability (Fig. 4K and Supplementary Fig. 5). Consistently, ectopic expression of Esg increases the number of tip cells but reduces the total number of ASP cells^5^ (Fig. 4J,L). This clearly suggests that Esg restricts EGFR signaling in the more distal cells by inducing tip-cell fate. In contrast, cells further away from the Bnl source either do not activate FGF/Btl signaling or activate the pathway at low levels, due to an Mmp2-mediated mechanism of lateral inhibition^5^. Consequently, there is either no, or low levels of transcription of *esg* in stalk/middle cells, allowing the full activation of EGFR signaling in the central part of the prospective air sac, and therefore enhancing cell division in this area (Fig. 4M). Nevertheless, our experiments do not exclude the possibility that Esg could regulate cell proliferation at the tip of ASP through another unknown process. Further studies are required to discard such possibility.

ASP development resembles that of vertebrate organs in that cell proliferation and cell death are concomitant with branching morphogenesis. Therefore, it is likely that the signaling crosstalk described here for the *Drosophila* ASP is also used in other developmental processes, in a comparable way. Accordingly, a similar modulation of EGFR signaling has been seen in the development of other tubular structures, such as the Malpighian tubules. In this case Wg signaling activates the expression of *rho* in both the tip and sibling cell in order to produce the ligand Spi, which in turn promotes cell division and cell survival^28^. In lung development, branch outgrowth and elongation are associated with cell division. In this tissue the coordinated activity of signaling factors including Wnts and Fgf10 regulate proliferation^29^,^30^,^31^. In the mammary gland, FGF signaling stimulates cell proliferation and/or survival to generate cells both at the branching epithelium tips and cells in the subtending duct^32^,^33^,^34^. Whether this mitotic effect is directly mediated by FGF or through another signaling pathway is still unknown. However, although no link between FGF and EGFR in mammary gland has yet been reported, it is tempting to speculate that the regulatory interplay between such signaling pathways might also promote branching and growth in vertebrates. Future studies should address the relationship of these pathways in branching development.

## Experimental Procedures

### Drosophila Strains and Genetics

Conditional activation of either RNAi or gene expression in the tracheal system was achieved by using the Gal4/Gal80^ts^ system^35^. *btlGAL4UASnGFP; tubulinGal80*^*ts*^ were crossed to the following UAS lines: *UASvn*^*RNAi*^ (#109437 and #50358), *UASpnt*^*RNAi*^ (#105390) and *UASesg*^*RNAi*^ (#9794) from VDRC; *UASspi*^*RNAi*^ (JF03322, HMS01120), *UASpnt*^*RNAi*^ (HMS01452), *UASesg*^*RNAi*^ (HMS00025) from Harvard Transgenic RNAi Project. *UASmae*, *UASp35* and *UASpntP2*^*VP16*^ from Bloomington Stock center; *UAS-esg*^36^, *UASvn*^37^, *UASaos*^38^, *UAStor-btl*^39^, *UASDER*^*DN*^^40^ and kept at 18 °C until late in L2 when larvae were shifted to 29 °C for 1–2 days and immediately dissected. For over-activation of FGF signaling in the trachea *dppGal4 UASGFP* (Bloomington) was crossed to *UASbnl*^41^.

The enhancer trap strain *vnGal4, UAS-GFPnls*^17^ and *vn*^*rF264*^*lacZ* reporter were used to visualize *vn* expression. *pnt*^*HS20*^ 18 and *pnt*^*1277*^to visualize *pntP1* and *pntP2* isoform expression respectively. *rholacZ* was used to monitor *rho* gene expression (Bloomington Stock Center).

### Flp-Out Clones

For clonal analysis, *hsflp; btl>y>Gal4; UAStauGFP* females were crossed with males carrying different UAS constructs listed above. Embryos were kept at 25 °C until late L2, incubated 1 hour at 37 °C and transferred to 25°C until late L3. Clone size was measured following the same criteria described in^3^.

### Immunohistochemistry

The following antibodies and dilutions were used: rat anti-Twist (gift from A. Stathopoulos, California Institute of Technology, Pasadena, USA), mouse anti-β-Gal (1:1,000, Sigma), mouse anti-Hnt (1:200) and anti-Spi (1:500, Developmental Studies Hybridoma Bank); rabbit anti-PH3 (1:1000) and mouse anti-Cas3 (1:1000, Cell signaling) and TRITC- or CY5-conjugated secondary antibodies (1:500; Jackson Immunoresearch). Rabbit and mouse secondary antibodies Alexa 555 and rat Alexa 647 (1:500; Invitrogen) were used. 546-labeled Phalloidin was incubated with the tissue for 20 min to visualize cell membrane. Samples were mounted in medium (Vectashield) with DAPI to stain the nucleus and analyzed by confocal microscopy (Leica SP5). Fixation and staining followed standard protocols.

## Additional Information

**How to cite this article**: Cruz, J. *et al.* FGF coordinates air sac development by activation of the EGF ligand Vein through the transcription factor PntP2. *Sci. Rep.* **5**, 17806; doi: 10.1038/srep17806 (2015).

## Supplementary Material

Supplementary Information
