# Author Correction: FGF coordinates air sac development by activation of the EGF ligand Vein through the transcription factor PntP2

**DOI:** 10.1038/s41598-022-16758-3

**Published:** 2022-08-03

**Authors:** Josefa Cruz, Neus Bota-Rabassedas, Xavier Franch-Marro

**Affiliations:** 1 Institute of Evolutionary Biology (IBE, CSIC-Universitat Pompeu Fabra), P. de la Barceloneta 37, 08003 Barcelona, Catalonia Spain; 2 Present Address: University of Texas MD Anderson Cancer Center, Houston, TX USA

Correction to: *Scientific Reports* 10.1038/srep17806, published online 03 December 2015

The Acknowledgements section in the original version of this Article is incomplete.

“We thank A. Simcox, A. Stathopoulos, B. Edgar, the Vienna Drosophila RNAi Center, the Bloomington Stock Center and the Developmental Studies Hybridoma Bank for flies and antibodies; K. Campbell, M. Llimargas, D. Martín and F. Wendler for helpful suggestions on the manuscript. J.C. was supported by BFU2009-08748 from the Spanish MICINN and N.B-R. was supported by FPI from the Spanish MICINN. This work was funded by the Spanish Ministerio de Ciencia e Innovacion (Project BFU2009-08748).”

should read:

“We thank A. Simcox, A. Stathopoulos, B. Edgar, the Vienna Drosophila RNAi Center, the Bloomington Stock Center and the Developmental Studies Hybridoma Bank for flies and antibodies; K. Campbell, M. Llimargas, D. Martín and F. Wendler for helpful suggestions on the manuscript. J.C. was supported by BFU2009-08748 from the Spanish MICINN and N.B-R. was supported by FPI from the Spanish MICINN. This work was funded by the Spanish Ministerio de Ciencia e Innovacion (Project BFU2009-08748) and Spanish Ministerio de Economía y Competitividad (Project CGL2014-55786-P). The research has also benefited from FEDER, UE funds.”

