# Supplementary material for: FGF coordinates air sac development by activation of the EGF ligand Vein through the transcription factor PntP2
Source: Sci Rep. 2015 Dec 3;5:17806. doi: 10.1038/srep17806 (PMC4668582; doi:10.1038/srep17806)
Supplement: Supplementary Information [file srep17806-s1.pdf]

# FGF coordinates air sac development by activation of the EGF ligand Vein through the transcription factor PntP2.

## Supplementary Information

Josefa Cruz*, Neus Bota-Rabassedas* and Xavier Franch-Marro†
Institute of Evolutionary Biology (IBE, CSIC-Universitat Pompeu Fabra), P. de la Barceloneta 37, 08003 Barcelona, Catalonia, Spain.

† Author for correspondence: xavier.franch@ibe.upf-csic.es
*These authors contributed equally to this work.

## Supplementary Information

Figure S1. Characterization and validation of the efficacy of the *vn* $^{RNAi}$*, spi*$^{RNAi}$ constructs and *UASaos* used in this paper. Two independent *RNAi* lines of each gene were over-expressed in the wing disc (*nubGAL4*) and in the tracheal system (*btlgal4UASGFP*) producing equivalent phenotypes. (A) Control adult wing and ASP. (B) Expression of *vn*$^{RNAi}$ in the wing disc and trachea produced smaller wings with vein defects and abrogated ASP formation. (C) Adult wing and ASP expressing *spi*$^{RNAi}$. Lack of *spi* has no effect in either the wing or the ASP development. (D) Over-expression of Aos resulted in a moderate EGFR phenotype like in adult wing and no effect in ASP. (E-F) ASP from a wild-type fly stained at the end of third instar for Spi (E-E') and *rholacZ* enhancer trap (F). (G) ASP visualized by Hnt in a wing disc over-expressing *vn*$^{RNAi}$ under the control of *apGAL4*.

Figure S2. Support and corroborate the findings of Figure 1. It shows the expression pattern of *vn* in ASP during early, mid and late L3 instar using *vnGal4UASGFP*. Arrowheads show *vn* expression in the wing disc.

Figure S3 expand the data presented in Figure 2 showing the specific effect of *vn* in ASP development. (A-B) *btlGAL4* tracheal mitotic cells marked with DAPi. Nucleai of the Dorsal Trunk cells of the second tracheal metamer (A) and spiracular branch cells (B). (C-D) DAPI staining of tracheal cells over-expressing *UASvn*$^{RNAi}$ under control of *btlGAL4.* Note that the number of tracheal mitotic Dorsal Trunk cells (C) and Spiracular branch cells (D) is unchanged.

Figure S4 expand the data presented in Figure 3. (A-B) *dppGAL4UASGFP UASbnl* wing discs showing a remarkable expansion of tracheal cells marked with Hnt (in blue) that expressed high levels of *vnlacZ* (in red). (C) Wild type ASP expressing GFP under the control of *btlgal4*. (D) ASP over-expressing *UASbnl* under the control of *btlGAL4UASGFP*. Note the increase of

tracheoblasts in the ASP.

Figure S5 extends the experiments presented in Figure 3. (A-F) Flip out clones over-expressing *tauGFP* (A), *UASDER$^{DN}$* (B), *UASvn$^{RNAi}$* (C), *UASpnt$^{RNAi}$* (D), *UAStor-btl* (E) and *UASvn* (F). The graphics display the percentage of clones found to extend to the tip (blue bars) and the distribution of their size (Red bars). n = number of air sac clones analyzed. Note that *UASDER$^{DN}$* and *UASvn$^{RNAi}$* expressing clones shown higher number of small clones and clones found at the tip (B and C) whereas *UAStor-btl* and *UASvn* produced more medium-large clones with low number of clones at the tip (E-F).

Figure S6. Corroborate the data presented in Figure 4. Characterization and validation of the efficacy of two independent *pnt $^{RNAi}$* lines in the adult fly wing, the adult eye and the ASP of the last instar larva. (A) Control adult wing. (B) *apGal4 UASpnt$^{RNAi}$* wing. (C) Control adult eye. (D) *eyelessGAL4/UASpnt$^{RNAi}$* adult fly eye . (E) *btlgal4UASGFP/UASpnt$^{RNAi}$* ASP. Note that the ASP is not formed in absence of *pnt.*

Figure S7. Support and corroborate the findings of Figure 4. (A-A'') *pntP2lacz* is expressed in both myoblast and tracheoblast of the wing disc. *btlGAL4/UASGFP* (green); *pntP2lacZ* (red); Twist (blue). The ASP is outlined with the white line. *pntP2lacZ* is detected in myoblasts and ASP cells, which also express the marker Twist (pink cells in merge) and tracheoblast, marked with GFP (yellow cells in merge). (B-B'') High magnification of the wing disc area, where ASP forms showing *pntP2lacz* expression in the tracheoblasts. Note that tracheoblasts do not express the myoblast marker Twist.

Figure S8. Support and expand the data presented in Figure 4. (A-A') Wing disc expressing *bnl* under the control of *dppGAL4UASGFP.* Ectopic expression of esg (in red) is detected in the tracheal cells. (B-C) Late L3 *btlGAL4UASGFP* control (B) and *esg$^{RNAi}$* (C) air sacs stained with anti-PH3.

(B' and C') PH3 staining only. The air sacs are outlined with the white line. Note the higher number of PH3 positive cells in (B') compare to (C'). (D) Cell number quantification of control and *esg*$^{RNAi}$ expressing ASP. The number of cells at the tip is significantly increased in *esg* deficient ASP cells. Error bars represent SEM (n = 8). Asterisks indicate differences statistically significant P ≤ 0.00006 (t test).

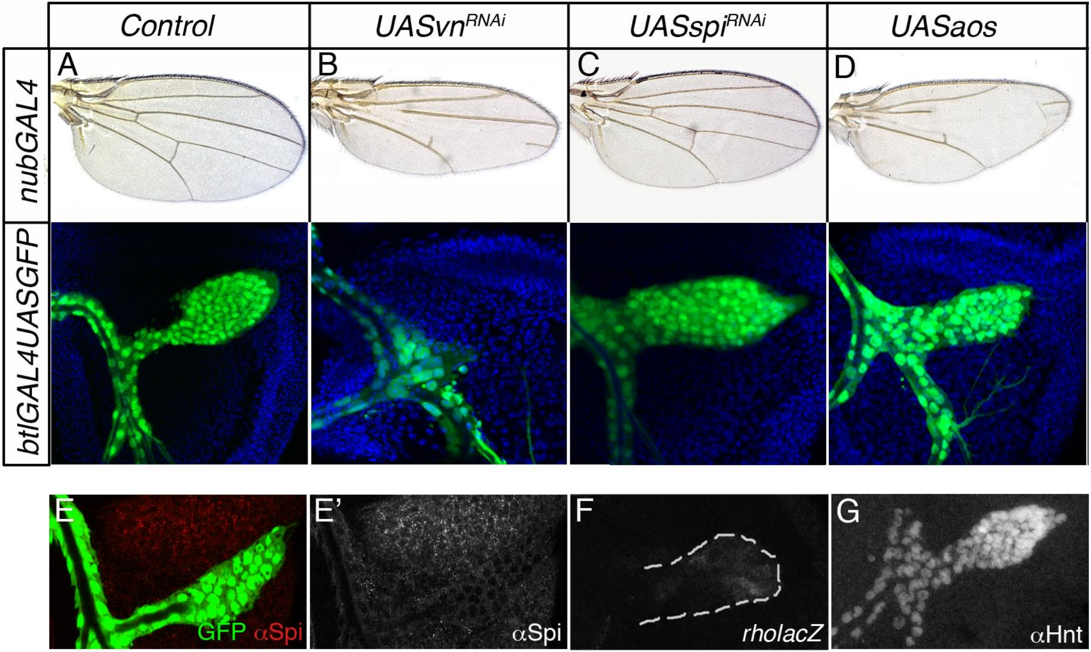
Control
UASvnRNAi
UASspiRNAi
UASaos
nubGAL4
btlGAL4UASGFP
A
B
C
D
E
E’
F
G
GFP αSpi
αSpi
rholacZ
αHnt

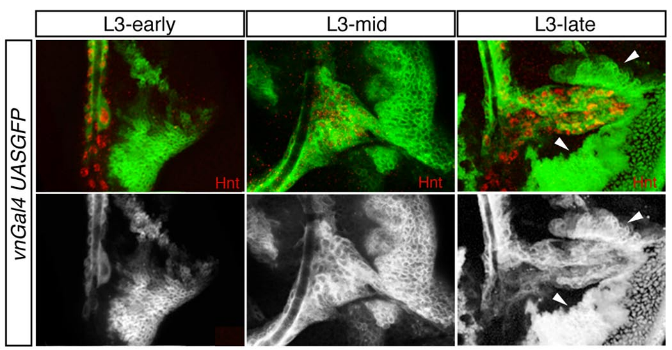
L3-early
L3-mid
L3-late
vnGal4 UASGFP
Hnt
Hnt
Hnt

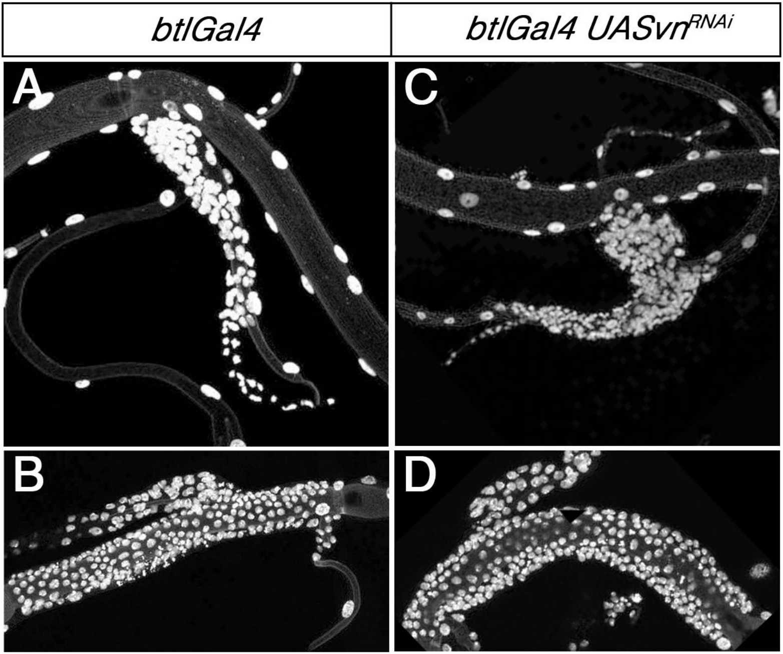

*btlGal4*
*btlGal4 UASvn^RNAi*
A
B
C
D

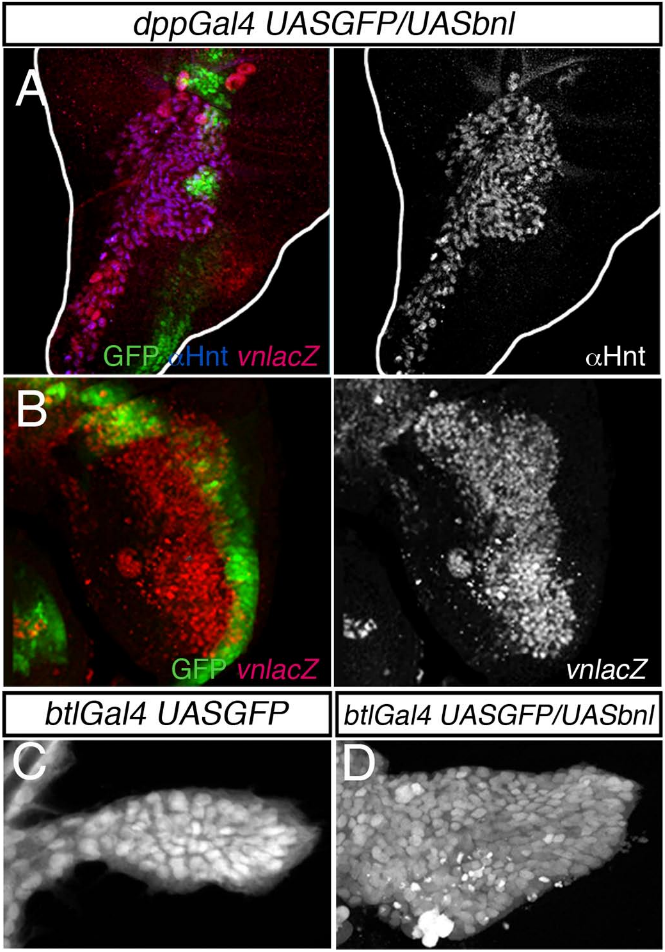
dppGal4 UASGFP/UASbnl
A
GFP αHnt vnlacZ
αHnt
B
GFP vnlacZ
vnlacZ
btlGal4 UASGFP
btlGal4 UASGFP/UASbnl
C
D

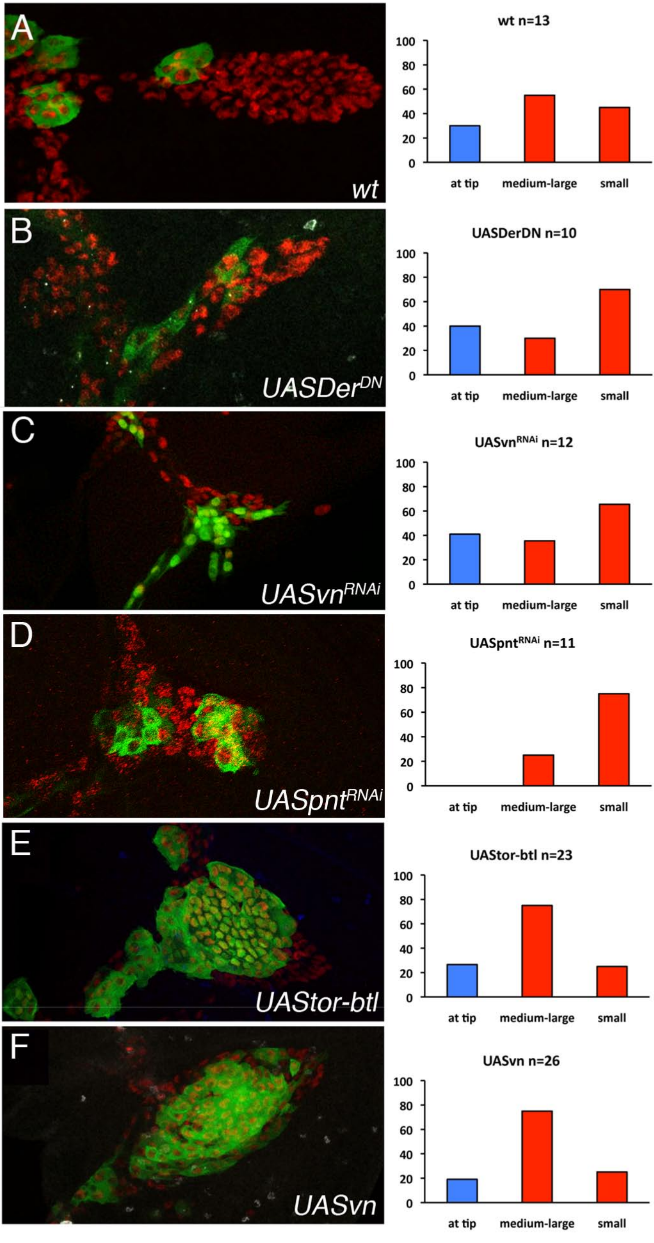
A
wt
wt n=13
100
80
60
40
20
0
at tip
medium-large
small
B
UASDer^DN
UASDerDN n=10
C
UASvn^RNAi
UASvn^RNAi n=12
D
UASpnt^RNAi
UASpnt^RNAi n=11
E
UAStor-btl
UAStor-btl n=23
F
UASvn
UASvn n=26

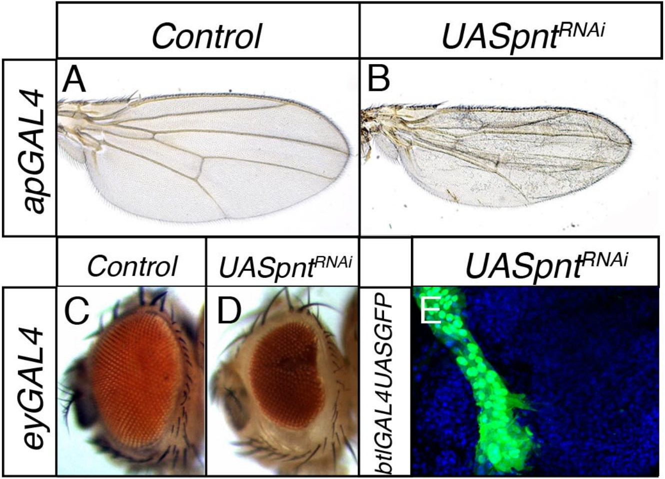
Control
UASpnt^RNAi
apGAL4
A
B
Control
UASpnt^RNAi
UASpnt^RNAi
eyGAL4
C
D
btlGAL4UASGFP
E

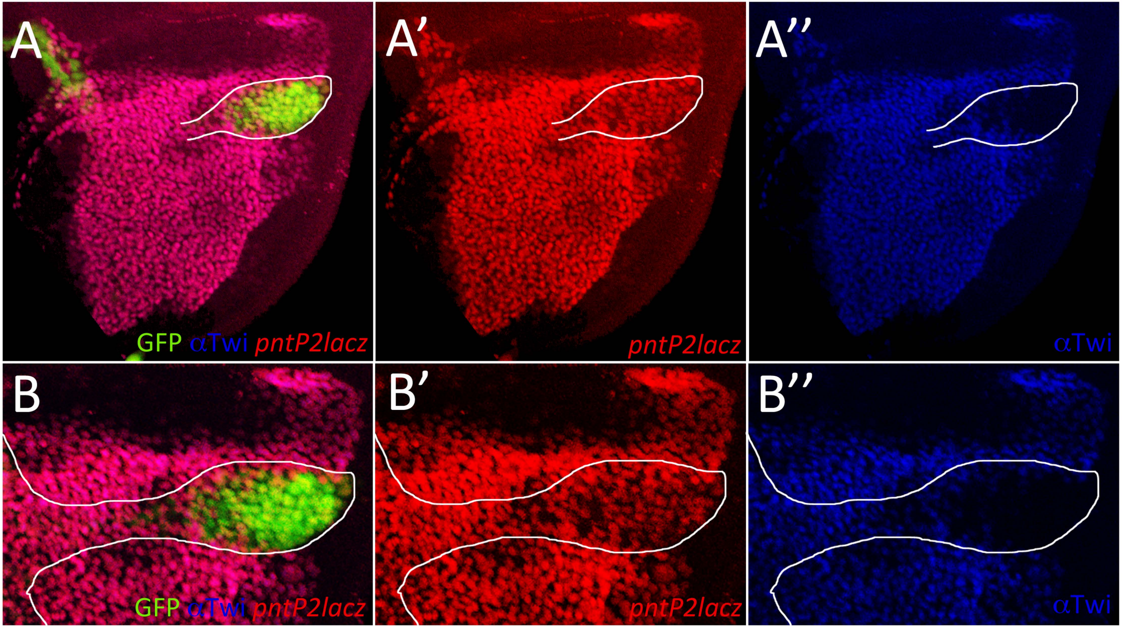
A
A’
A’’
GFP αTwi *pntP2lacz*
*pntP2lacz*
αTwi
B
B’
B’’
GFP αTwi *pntP2lacz*
*pntP2lacz*
αTwi

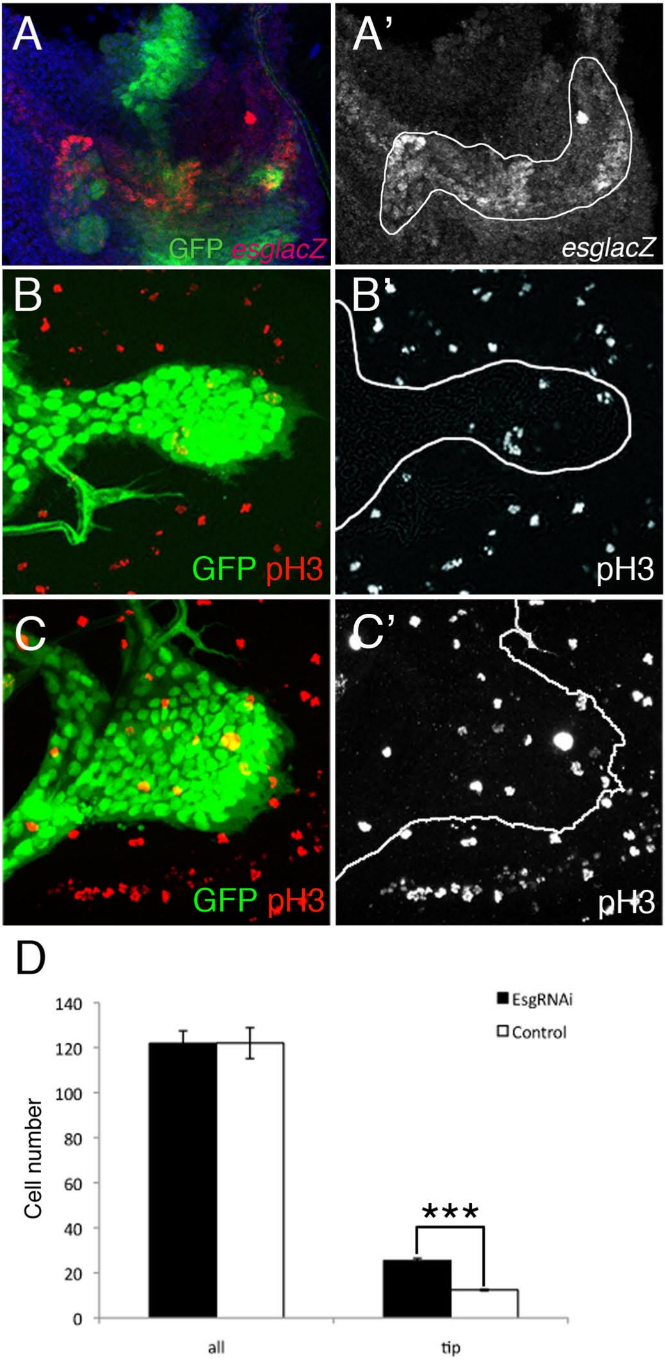
A
A'
GFP esglacZ
esglacZ
B
B'
GFP pH3
pH3
C
C'
GFP pH3
pH3
D
EsgRNAi
Control
Cell number
140
120
100
80
60
40
20
0
***
all
tip
